# Validity of forensic cartridge-case comparisons

**DOI:** 10.1073/pnas.2210428120

**Published:** 2023-05-08

**Authors:** Max Guyll, Stephanie Madon, Yueran Yang, Kayla A. Burd, Gary Wells

**Affiliations:** ^a^School of Social and Behavioral Sciences, Arizona State University, Glendale, AZ 85306; ^b^Department of Psychology, University of Nevada-Reno, Reno, NV 89557; ^c^Department of Psychology, University of Wyoming, Laramie, WY 82071; ^d^Department of Psychology, Iowa State University, Ames, IA 50011

**Keywords:** forensic examination, cartridge-case comparisons, validity, inconclusive decisions, probative value

## Abstract

This article presents key findings from a research project that evaluated the validity and probative value of cartridge-case comparisons under field-based conditions. Decisions provided by 228 trained firearm examiners across the US showed that forensic cartridge-case comparison is characterized by low error rates. However, inconclusive decisions constituted over one-fifth of all decisions rendered, complicating evaluation of the technique’s ability to yield unambiguously correct decisions. Specifically, restricting evaluation to only the conclusive decisions of identification and elimination yielded true-positive and true-negative rates exceeding 99%, but incorporating inconclusives caused these values to drop to 93.4% and 63.5%, respectively. The asymmetric effect on the two rates occurred because inconclusive decisions were rendered six times more frequently for different-source than same-source comparisons. Considering probative value, which is a decision’s usefulness for determining a comparison’s ground-truth state, conclusive decisions predicted their corresponding ground-truth states with near perfection. Likelihood ratios (LRs) further showed that conclusive decisions greatly increase the odds of a comparison’s ground-truth state matching the ground-truth state asserted by the decision. Inconclusive decisions also possessed probative value, predicting different-source status and having a LR indicating that they increase the odds of different-source status. The study also manipulated comparison difficulty by using two firearm models that produce dissimilar cartridge-case markings. The model chosen for being more difficult received more inconclusive decisions for same-source comparisons, resulting in a lower true-positive rate compared to the less difficult model. Relatedly, inconclusive decisions for the less difficult model exhibited more probative value, being more strongly predictive of different-source status.

## Significance

Comprehensive evaluation of a forensic technique's validity should entail consideration of not only error rates, but accuracy and inconclusive rates, as well. The fact that a technique excels at avoiding errors does not ensure that it is equally successful at reaching correct decisions. This research, which focused on cartridge-case comparison, showed that error rates were uniformly low, but other performance measures varied by the firearm model and the treatment of inconclusive decisions. Importantly, inconclusive decisions occurred frequently and predicted different-source status, the ground-truth state supportive of factual innocence. Mindful of the probative and exculpatory value of inconclusive decisions, prosecutors should disclose inconclusive results to the defense, and defense attorneys should consider inconclusive results as a possible basis for post-conviction actions.

The validity of forensic techniques has come under heavy scrutiny. In 2009, the National Research Council identified significant deficits in scientific knowledge regarding a broad range of forensic techniques (1). In 2016, the President’s Council of Advisors on Science and Technology (PCAST) provided an updated assessment of the situation that reiterated many of the original concerns (2). Both reports called for scientific assessments of the validity of forensic techniques to determine whether they are founded upon sound principles and methods, as evidenced by their ability to generate accurate decisions. In response to these calls, we report the findings of a large-scale validity study involving 228 firearm examiners who performed 1,811 microscopic comparisons of fired cartridge cases, a commonly used forensic technique (3). All examiners were employed in private, municipal, county, state, or federal crime laboratories across the United States at the time of their participation. To advance understanding about the accuracy of cartridge-case comparisons under field-based conditions, this research used firearms that had been in circulation in the general population, examined technique performance with respect to two dissimilar firearm models, and employed an open-set design.

## Cartridge-Case Comparison

A primary goal of cartridge-case comparison is to determine the ground-truth state of two or more cartridge cases; that is, to determine whether the cartridge cases have been fired from the same firearm (same-source status) or from different firearms (different-source status). A corresponding task in casework could entail an examiner comparing a “questioned” cartridge case recovered from a crime scene to “known” cartridge cases fired from a suspect’s firearm to determine if the suspect’s firearm also fired the questioned cartridge case. To begin, examiners first compare the cartridge cases with regard to a firearm’s class characteristics, such as caliber and firing pin aperture shape. If all class characteristics match, examiners next compare the cartridge cases with regard to toolmarks produced by a firearm’s individual characteristics, which include microscopic imperfections that arise from the manufacturing process, as well as from a firearm’s use and maintenance.

A firearm’s individual characteristics are highly distinctive and the profession asserts them to be unique (4). Therefore, sufficient agreement of toolmarks produced by individual characteristics supports the conclusion that two cartridge cases were fired from the same firearm, leading to an identification decision, whereas sufficient disagreement supports the conclusion that the cartridge cases were fired from different firearms, leading to an elimination decision. If there is neither sufficient agreement nor sufficient disagreement of the toolmarks, then an unambiguous source determination decision cannot be made, leading to an inconclusive decision (5). These decision categories are the same as those for bullet and general toolmark comparison, and conceptually similar to those used by a variety of other forensic techniques, such as fingerprint comparison (6) and polygraph examination (7).

## Technique Validity

Although the forensic science literature includes a number of empirical studies that have sought to test the validity of cartridge-case comparison, nearly all are characterized by methodological flaws, most notably a reliance on a closed-set design (2). In a closed-set design each cartridge case can be matched to another that has been fired from the same firearm. Because every cartridge case has a match, the comparisons are not independent, and the task is simpler than that associated with actual casework wherein a cartridge case may not have a match. In other words, a closed-set design only requires examiners to find the cartridge case that most closely matches another cartridge case to render a correct identification decision, a strategy that would be ineffective and inappropriate in the field. Thus, a closed-set design is ill-suited to establishing validity because it potentially overestimates accuracy and underestimates error (2, 8).

Only two large sample validity studies of cartridge-case comparison have avoided the limitation of a closed-set design. One study that collected data from 218 firearm examiners (9) reported a false-positive rate of 1.0% and a false-negative rate of 0.4%. Although this was a methodologically strong study, several factors could have affected its results. The study did not vary the number of same-source versus different-source comparisons between examiners, which could have assisted examiner decision making via cross-talk. The study used only a single firearm model to produce the cartridge cases, none of the firearms had previously been used, and many had been manufactured proximally to others, factors that could have affected the representativeness and difficulty of the comparison task relative to that which is typically encountered in casework. It also bears mentioning that the study did not undergo peer review, which is the primary means by which the scientific community ensures that published research is of high quality and thus is one of PCAST’s criteria for scientific rigor. In the second study (10) researchers purposefully chose firearms and ammunition with the intent of producing comparisons that examiners would find to be challenging. This methodologically sound and peer-reviewed study yielded a false-positive rate of 0.9% and a false-negative rate of 1.8%. Taken together, results of these two studies suggest that error rates associated with cartridge-case comparison may generally be low.

## Treatment of Inconclusives in Calculating Error Rates

Having a low error rate is an important factor to be considered in a court’s ruling on the admissibility of forensic evidence and expert forensic testimony. Specifically, part of the admissibility ruling hinges on determining whether the technique used to inform forensic expert testimony is based on scientifically valid principles and reasoning. In making this determination, the US Supreme Court’s Daubert opinion states that a judge “…ordinarily should consider the known or potential rate of error…” (11). Calculating a technique’s error rate would be straightforward if the technique only permitted examiners to make identification and elimination decisions, each of which must be either correct or incorrect with respect to ground truth. However, as noted above, cartridge-case comparison and other forensic techniques also permit examiners to make inconclusive decisions, which raises the question as to how inconclusives ought to be treated when calculating error rates.

Uncertainty regarding the treatment of inconclusives has led to consideration of a number of approaches. For example, using data from prior firearm examination studies, researchers (12) have calculated potential error rates while treating all inconclusives as either correct decisions (i.e., correctly reflecting examiner uncertainty), as incorrect decisions (i.e., reflecting a procedural failure to make a conclusive decision of identification or elimination) (13), or as equivalent to eliminations (i.e., viewing both elimination and inconclusive decisions as failures to make an identification). Error rate estimates varied widely depending on the chosen treatment, which is to be expected given that inconclusive decisions are a common forensic result.

Other scholars contend that in characterizing forensic decisions as correct or incorrect, each decision warrants individualized treatment that involves evaluating whether its corresponding comparison provided sufficient information to justify a conclusive decision (14). One proposal entails basing a sufficiency determination on the consensus opinion of some designated body, such as a panel of expert examiners (15, 16). According to this approach, an inconclusive decision would be deemed incorrect if the designated body viewed the comparison as providing sufficient information to justify a conclusive decision. This approach further proposes that the designated body may deem inconclusive to be the correct decision if it is of the opinion that there was insufficient information to justify a conclusive decision, in which case identifications and eliminations would be deemed incorrect. Critics of this approach highlight its potential to yield illogical outcomes, such as considering a conclusive decision to be incorrect even when it is correct with regard to ground truth (17, 18). Moreover, consensus opinions regarding the sufficiency of information for making a forensic decision could vary by the individuals providing the opinion and could even vary among the same individuals over time (19–21), either of which could cause a particular decision to be judged as correct on one occasion but as incorrect on another. Particularly worrisome is the potential for the consensus opinion itself to be conclusive but incorrect with regard to ground truth (22). Another proposal suggests making the sufficiency determination by means of a computer algorithm that models the similarity of same-source and different-source comparisons (8). Although an algorithm has the benefit of consistency, it is still vulnerable to illogical outcomes and likewise rests on human opinion both to choose the similarity measure and to set the criterion values at which a decision would switch between being judged as correct versus incorrect.

Whereas the treatments described above all involve human subjectivity, the conventional approach of determining a decision’s correctness on the basis of its correspondence with ground truth uses a criterion that is objective, factual, and immutable (18). For identification and elimination decisions, the determination of decision correctness is unambiguous because both decisions categorically assert a single ground-truth state that must be either correct or incorrect. Inconclusive decisions, however, are different because they expressly forgo any assertion as to the ground-truth state of the evidence. From this perspective, inconclusives can be neither correct nor incorrect, meaning that they constitute a special kind of decision that is fundamentally different from identifications and eliminations (23).

One way to account for the special status of inconclusive decisions is to simply omit inconclusive decisions and calculate accuracy and error rates on the basis of identification and elimination decisions alone. When inconclusive decisions are omitted from the calculations, accuracy and error rates sum to one, thereby creating a situation in which error rates alone fully characterize a technique’s performance. However, by omitting inconclusives this approach ignores a common forensic decision that the forensic profession considers to be fully legitimate (5, 24). Furthermore, omitting inconclusives can skew accuracy and error rates by basing them on only a subset of comparisons evaluated, essentially treating inconclusives as if they did not occur (25). By way of an example, if inconclusives are omitted from calculations, the 1% false-positive rate from research referenced above (9) implies a true-negative rate of 99%. However, examiners in that study actually correctly detected different-source status in only 65% of all different-source comparisons evaluated, a substantial reduction reflecting the fact that 34% of different-source comparisons received inconclusive decisions.

A second way to account for the special status of inconclusives is to examine them as legitimate decisions in their own right and to incorporate them into accuracy and error rate calculations (17). This approach removes the redundancy between error and accuracy, causing error rates to become inadequate as sole indicators of validity. Incorporating inconclusives will only minimally affect error rates that would otherwise be quite low, but can substantially reduce accuracy rates that would otherwise be quite high. Inconclusives can also have an uneven effect on the two accuracy rates. That is, if inconclusives are more frequently assigned to either same-source or different-source comparisons, then either the true-positive rate or the true-negative rate will be more greatly reduced.

## Probative Value

Evaluations of a technique’s validity typically entail calculating the probability of a particular forensic decision given a particular ground-truth state. However, for evidence to be admitted in court, not only must the technique on which it is based be scientifically valid, but the evidence it yields must also have probative value, meaning that the forensic decision will “make a fact more or less probable” (26), and help the trier of fact “to determine a fact in issue” (27). Thus, probative value reflects the converse conditional probability; specifically, the probability of a particular ground-truth state given a particular forensic decision (12, 28). In other words, the purpose of a forensic decision in the context of casework is to provide a predictive indicator of ground truth, the latter of which is unknown. It is critically important, therefore, to evaluate the extent to which forensic decisions possess probative value.

### Predictive Value.

The positive and negative predictive values (PPV and NPV) provide metrics of the predictive relationship between conclusive decisions and ground truth (29). In short, they represent the probability that a conclusive decision is correct. With respect to cartridge cases, PPV equals the proportion of all comparisons given identification decisions that are truly same-source (i.e., the proportion of identification decisions that are correct), and NPV equals the proportion of all comparisons given elimination decisions that are truly different-source (i.e., the proportion of elimination decisions that are correct):

[1] PPV = p(same source|identification),

[2] NPV = p(different source|elimination).

Given that the term “predictive value” denotes decision correctness, it cannot be applied to inconclusive decisions because, as noted above, inconclusives can be neither correct nor incorrect with respect to ground truth. Nonetheless, inconclusive decisions could be associated with one ground-truth state more than the other, making them predictive of ground truth, and therefore informative in practice. PPV, NPV, and inconclusive decisions’ predictive relationship with ground truth depend on the underlying prevalence of same-source versus different-source comparisons (28). Because the prevalence value is unknown in the field, PPV, NPV, and inconclusive decisions’ predictive relationship with ground truth cannot be definitively determined in casework, but can be calculated as functions of prevalence.

### Likelihood Ratio (LR).

The LR is an alternative metric for linking forensic decisions to ground truth. Unlike predictive values, the LR has the desirable characteristic that it does not vary by prevalence. A LR can be calculated for all decisions—inconclusives as well as identifications and eliminations (30). The LR value reflects the decision’s support for the hypothesis that the evidence samples came from the same source relative to its support for the hypothesis that they came from different sources. A decision’s LR is calculated as the portion of all same-source comparisons that are given a particular decision divided by the proportion of all different-source comparisons that are given that same decision:

[3] $$\mathrm{LR}\;=\;\frac{p(\text{decision|same source})}{p(\text{decision|different source})}.$$

Thus, LR values can range from 0 to infinity, with values of 1 representing equivalent support for both ground-truth state hypotheses. As a decision’s LR increases above 1, that decision increasingly supports the same-source hypothesis; as it decreases below 1, that decision increasingly supports the different-source hypothesis. The pretest odds of the evidence samples being same-source (i.e., prior to learning the forensic decision) can be multiplied by the LR to give the posttest odds of the evidence samples being same-source (i.e., after learning the forensic decision):

[4] $$\begin{array}{c} {\text{posttest odds}}_{\text{same}\text{-source}}={\text{pretest odds}}_{\text{same}\text{-source}}\times \text{LR}. \end{array}$$

In corresponding fashion, the reciprocal of LR reflects a decision’s support for the different-source hypothesis relative to its support for the same-source hypothesis and communicates parallel information regarding ground-truth status:

[5] $$\frac{1}{\text{LR}}=\frac{p(\text{decision|different source})}{p(\text{decision|same source})}\text{,}$$

[6] $$\begin{array}{c} {\text{posttest odds}}_{\text{different}\text{-source}}\;=\;{\text{pretest odds}}_{\text{different}\text{-source}}\times \frac{1}{\text{LR}}. \end{array}$$

Eqs. **4** and **6** indicate the utility of LRs to triers of fact in the legal system, such as judges and juries, who are tasked with evaluating forensic decisions. Specifically, a trier of fact can directly take a decision’s LR as the posttest odds of the evidence being same-source if pretest odds equal 1, a situation corresponding to being initially unbiased and withholding all judgment as to a comparison’s ground-truth status prior to hearing the forensic decision. Values for 1/LR are similarly informative regarding different-source status.

Finally, the odds of either ground-truth state can be converted both to the odds of the other ground-truth state and to the probabilities of both ground-truth states through rearrangement of the mathematical relationships that link these quantities:

[7] $$\begin{array}{c} {\text{odds}}_{\text{same}\text{-source}}=\frac{1}{{\text{odds}}_{\text{different}\text{-source}}}=\frac{p(\text{same source})}{\text{1-}\text{p}(\text{same source})}\text{,} \end{array}$$

[8] 1=p(same source)+p(different source).

## Toolmark Variation and Validity

In performing cartridge-case comparisons, firearm examiners base their decisions on toolmarks transmitted by a firearm onto a cartridge case. However, the number, type, clarity, and consistency of the transmitted toolmarks can vary as a result of the different designs and manufacturing processes associated with different firearm models (31). Firearm model, therefore, represents a factor that could affect comparison difficulty and thereby affect examiners’ ability to make correct decisions. Accordingly, it is conceivable that an accuracy or error rate for the technique may not be represented by a single value, but instead might vary depending on the particular firearm model that fired the cases. The fact that firearm identification requests are made for a broad range of firearm models points to the importance of considering whether variation in toolmark characteristics associated with firearm model affects validity estimates for the technique and how the treatment of inconclusive decisions might moderate those results, a question that prior research has not addressed.

## Research Overview

This article presents data from a cartridge-case comparison validity study involving a large sample of forensic firearm examiners. The research advances scientific knowledge regarding forensic cartridge-case comparison by addressing three issues. First, it examined how omitting versus incorporating inconclusive decisions in performance measure calculations affected evaluation of the technique’s validity with respect to yielding unambiguously correct and incorrect identification and elimination decisions. Second, it compared results across two firearm models to examine the influence of variation in toolmark characteristics on examiners’ decisions, comparing results while both omitting and incorporating inconclusive decisions. Third, it examined the probative value of examiner decisions for determining ground truth by calculating the PPV, NPV, and inconclusive decisions’ predictive relationship with ground truth across possible prevalence values, as well as by calculating LR values for each forensic decision.

## Results

### Decision Frequencies.

Table 1 presents the frequencies of identification, elimination, and inconclusive decisions broken down by the firearm model and ground-truth status.

**Table 1.** Decision frequencies by firearm model and ground-truth status

	Forensic decision		
	Identification	Elimination	Inconclusive
Beretta 92FS			
Same-source	397	1	56 {=1^*^ +36^†^+18^‡^ + 1^§^}
Different-source	1	272	178 {=6^*^ +11^†^ +69^‡^ + 92^§^}
HiPoint C9			
Same-source	454	0	3 {=0^*^ +2^†^ + 1^‡^ + 0^§^}
Different-source	4	300	145 {=3^*^ +16^†^+17^‡^ +109^§^}

^*^Inconclusive without further characterization.

^†^Inconclusive with some agreement of individual characteristics but insufficient for identification.

^‡^Inconclusive without agreement or disagreement of individual characteristics.

^§^Inconclusive with disagreement of individual characteristics but insufficient for elimination.

Note: These data and all analyses omit the single decision of “Unsuitable for analysis” that occurred for a Beretta different-source comparison.

### Analytic Approach.

Examination of response distributions (*SI Appendix*, Figs. S1–S4) revealed that some examiners frequently reported inconclusive decisions for different-source comparisons, whereas others rarely or never did so. These bimodal data distributions show that decision probabilities varied across examiners and that the data exhibited nonindependence stemming from the multilevel structure of the data, wherein each examiner provided decision-level data for eight comparisons. These data characteristics violate assumptions of binomial-distribution-based analyses, precluding their use. We therefore estimated rates, rate differences, and CIs using a multilevel bootstrapping method based on 10,000 samples randomly drawn with replacement (32).

### Accuracy and Error.

Combining data for both firearm models, we estimated overall accuracy and error rates, first omitting and then incorporating inconclusive decisions. We scored each conclusive decision (i.e., identifications and eliminations) as correct if the decision’s assertion as to the comparison’s ground-truth state matched the comparison’s actual ground-truth state, and as incorrect if it did not. Because inconclusive decisions do not assert a ground-truth state, our use of a decision’s correspondence with actual ground-truth as the criterion for scoring correctness precluded scoring inconclusive decisions as either correct or incorrect.

#### Omitting inconclusive decisions.

Analyses first omitted inconclusive decisions and based accuracy and error rate estimates solely on decisions of identification and elimination, yielding two accuracy rates (true-positive rate; true-negative rate) and two error rates (false-negative rate; false-positive rate). Because this analysis omits inconclusive decisions, the true-positive and false-negative rates are redundant as are the true-negative and false-positive rates (i.e., true-positive rate + false-negative rate = 100%; true-negative rate + false-positive rate = 100%). As reported in the upper portion of Table 2, results indicated both a very high true-positive rate, indicating a high degree of sensitivity, and a very high true-negative rate, indicating a high degree of specificity. Consequently, the results equally revealed a very low false-negative rate and a very low false-positive rate. With regard to errors, the 1,429 conclusive decisions included a total of one false-negative and five false-positives. No single examiner made more than one error.

**Table 2.** Accuracy and error rates when omitting and incorporating inconclusive decisions

	Aggregate	Beretta 92FS	HiPoint C9
	*M*[95% CI]	*M*[95% CI]	*M*[95% CI]
	Omitting inconclusive decisions		
True-positive rate	0.999[0.995,1.000]	0.998[0.989,1.000]	1.000[0.992,1.000]^*^
True-negative rate	0.991[0.979,1.000]	0.996[0.983,1.000]	0.987[0.966,1.000]
False-negative rate	0.001[0.000,0.005]	0.002[0.000,0.011]	0.000[0.000,0.008]^*^
False-positive rate	0.009[0.000,0.021]	0.004[0.000,0.017]	0.013[0.000,0.034]
	Incorporating inconclusive decisions		
True-positive rate	0.934[0.908,0.958]	0.874[0.825,0.919]	0.993[0.981,1.000]
True-negative rate	0.635[0.579,0.691]	0.603[0.532,0.670]	0.668[0.600,0.734]
False-negative rate	0.001[0.000,0.005]	0.002[0.000,0.010]	0.000[0.000,0.008]^*^
False-positive rate	0.006[0.000,0.013]	0.002[0.000,0.011]	0.009[0.000,0.023]
Same-source inconclusive rate	0.065[0.041,0.091]	0.124[0.080,0.172]	0.007[0.000,0.019]
Different-source inconclusive rate	0.359[0.304,0.415]	0.395[0.328,0.467]	0.324[0.257,0.393]

^*^Calculated using the Clopper–Pearson method due to 0% incorrect decision rate, which would have precluded variation in bootstrap estimates.

Note: Mean estimates and confidence intervals calculated through a multi-level boostrapping method.

#### Incorporating inconclusive decisions.

Of all 1,811 comparisons, examiners rendered 382 inconclusive decisions for an overall inconclusive rate of 21.1%. This nontrivial inconclusive rate indicates the importance of considering how incorporating inconclusive decisions affects accuracy and error rates (12, 17). Incorporating inconclusive decisions increases the total number of comparisons under consideration, thereby decreasing accuracy and error rates alike, as reported in the lower section of Table 2. For accuracy rates, incorporating inconclusive decisions reduced the true-positive rate, or sensitivity, from 99.9% to 93.4%, and substantially reduced the true-negative rate, or specificity, from 99.1% to 63.5%. Error rates—which were already quite low at less than 1%—evidenced slight decreases.

To examine the effect of ground-truth status on producing inconclusive decisions, we tested the null hypothesis of no-difference in the proportions of inconclusive decisions among same-source and different-source comparisons. Results supported rejection of the null hypothesis and supported the alternative hypothesis that ground-truth state does affect the likelihood of an inconclusive decision. Specifically, examiners rendered inconclusive decisions less frequently for same-source than different-source comparisons (same-source inconclusive rate = 6.5%; different-source inconclusive rate = 35.9%; |difference| = 29.5%, CI_95%_ [23.8%; 35.3%], *p* < .001). This asymmetric assignment of inconclusive decisions to different-source comparisons underlies the large decrease in the true-negative rate when incorporating inconclusive decisions. These results show that a substantial percentage of different-source comparisons received inconclusive decisions rather than unambiguously correct elimination decisions. We return to this important observation in the *Discussion*.

### Effect of Firearm Model.

Cartridge cases fired from Berettas typically present fewer toolmarks which results in more difficult comparisons than cartridge cases fired from HiPoints (*SI Appendix*, Figs. S5 and S6), a factor that could affect examiner performance. Therefore, we tested the null hypothesis of no-difference between these firearm models in accuracy and error rates. We compared the models’ accuracy and error rates first while omitting and then while incorporating inconclusive decisions. We calculated each rate difference by subtracting the rate for the HiPoint model from the rate for the Beretta model. Table 2 presents all rates for each firearm model.

#### Omitting inconclusive decisions.

When considering only the conclusive decisions of identification and elimination, the accuracy rates did not statistically differ across the firearm models (true-positive rate_difference_ = 0.2%, CI_95%_ [0.0%; 1.1%], *p* > .05; true-negative rate_difference_ = −0.9%, CI_95%_ [−3.2%; 1.0%], *p* > .05). Because omitting inconclusive decisions creates redundancies between accuracy and error rates, firearm model effects on the false-negative rate and false-positive rate mirrored those on the true-positive rate and true-negative rate, respectively.

#### Incorporating inconclusive decisions.

Analyses testing firearm model effects when incorporating inconclusives revealed significantly less sensitivity for cartridge-case comparisons fired by the Beretta model than by the HiPoint model (true-positive rate_difference_ = −11.9%, CI_95%_ [−16.7%; −7.5%], *p* < .05), but no significant difference in specificity between the firearm models (true-negative rate_difference_ = −6.5%, CI_95%_ [−14.4%, 1.6%], *p* > .05). In addition, the Beretta and HiPoint error rates were quite similar and did not differ (false-negative rate_difference_ = 0.2%, CI_95%_ [0.0%; 1.0%], *p* > .05; false-positive rate_difference_ = −0.7%, CI_95%_ [−2.7%; 0.6%], *p* > .05). Comparing inconclusive decision rates across firearm models showed that examiners rendered inconclusive decisions at a significantly greater rate for Berettas than HiPoints when evaluating same-source comparisons (same-source inconclusive rate_difference_ = 11.7%, CI_95%_ [7.4%; 16.5%], *p* < .05). The same pattern characterized different-source comparisons, although the difference did not achieve statistical significance (different-source inconclusive rate_difference_ = 7.2%, CI_95%_ [−1.0%; 15.2%], *p* > .05).

### Probative Value.

#### Predictive values and inconclusives’ prediction of ground truth.

PPV, NPV and inconclusive decisions’ predictive relationship with ground truth depend not only on the accuracy, error, and inconclusive rates reported above, but also on the prevalence of the ground-truth state or, in other words, the proportion of comparisons that are same-source. Although a single value for each decision’s relationship with ground truth could be calculated at the 50% prevalence rate used in the present study, the results would only reflect values to be expected in the field if prevalence in the field were also 50%. Because field prevalence is not known, we calculated predictive values across all possible prevalence values.

**Fig. 1.**
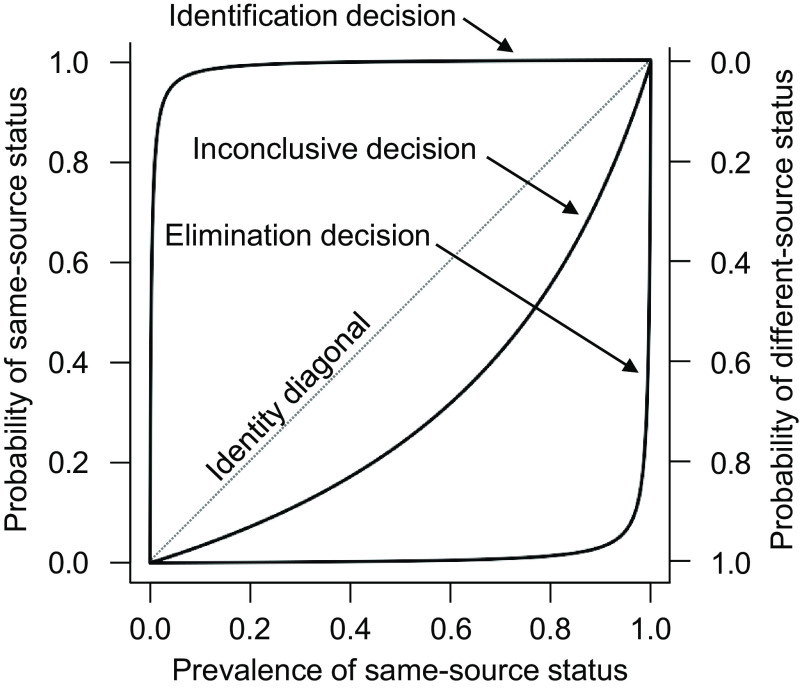
Decisions’ prediction of ground-truth status for Beretta 92FS.

**Fig. 2.**
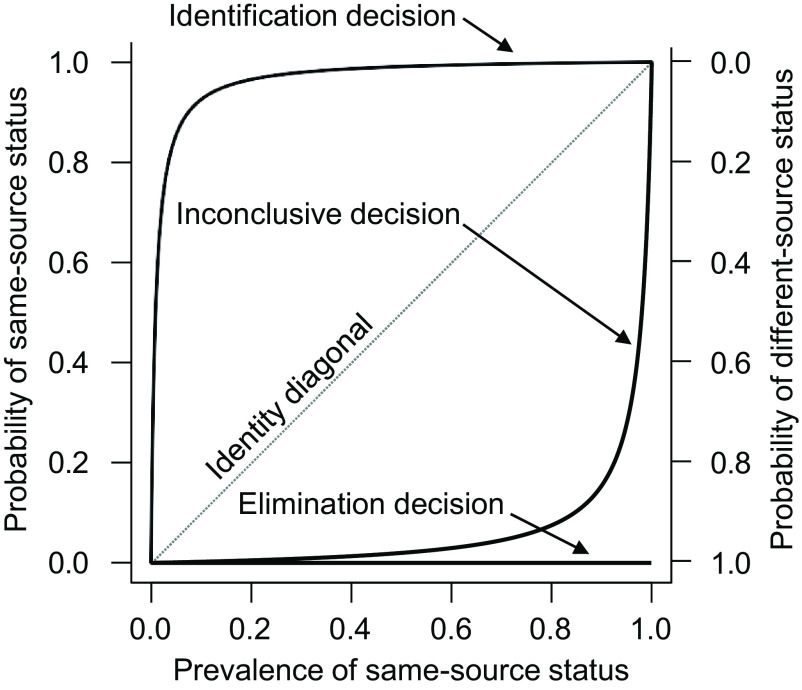
Decisions’ prediction of ground-truth status for HiPoint C9.

Figs. 1 and 2 present the forensic decisions’ predictive relationships with ground truth for decisions rendered for cartridge cases fired from the Beretta and HiPoint model, respectively. Each figure presents three curves, one each for identification, elimination, and inconclusive decisions. The identity diagonal of each graph depicts where a predictive relationship would equal the prevalence value. If a decision’s curve is on the identity diagonal, then that decision possesses no ability to truly discriminate between same-source and different-source comparisons. To illustrate, imagine if examiners never bothered to actually examine any comparison, but instead simply reported “identification” for each and every comparison. Even in this absurd scenario the PPV would equal the prevalence of same-source comparisons, thereby illustrating that the more the curve for a particular decision departs vertically from the identity diagonal, the more the assignment of that decision truly discriminates between the ground-truth states. Thus, the further the curve is above the diagonal, the more strongly the corresponding decision meaningfully predicts same-source status, and the further the curve is below the diagonal, the more strongly it meaningfully predicts different-source status. The curves are forced to the diagonal at prevalence values of 0% and 100% where only a single ground-truth state exists, making it impossible for a forensic decision to discriminate between the ground-truth states because ground-truth status does not vary.

Figs. 1 and 2 show that for both firearms, identification decisions were highly predictive of same-source status and elimination decisions were highly predictive of different-source status across all but the most extreme prevalence values. The figures further reveal that inconclusive decisions showed a clear tendency to predict different-source status, in that the curves for inconclusive decisions depart vertically downward from the diagonal. Moreover, inconclusive decisions’ predictive relationship with ground truth appeared to differ between the two firearm models. Inconclusive decisions tended to predict different-source status particularly strongly for comparisons produced by the HiPoint model. For example, if prevalence were assumed to equal 50% such that same-source and different-source status were equally likely, then 76.2% of inconclusive decisions rendered for Beretta comparisons would be different-source, whereas 98.0% of inconclusive decisions rendered for HiPoint comparisons would be different-source.

#### Likelihood ratios.

The LR quantifies how much learning of a forensic decision changes the odds of a comparison’s ground-truth status being same-source. Therefore, the LR reflects a decision’s support for the same-source hypothesis. Alternatively, 1/LR provides corresponding information for the different-source hypothesis. Table 3 presents values for both LR and 1/LR for each decision as estimated from the aggregate data and from the data specific to each firearm model.

**Table 3.** Decision likelihood ratios

	Aggregate		Beretta 92FS		HiPoint C9	
	*Median*	[95% CI]	*Median*	[95% CI]	*Median*	[95% CI]
	LR^*^					
Identification	177.458	[71.210, >1000]	>1000	[88.792, >1000]	118.976	[44.041, >1000]
Elimination	<0.001	[<0.001, 0.008]	<0.001	[<0.001,0.017]	<0.001	[<0.001, <0.001]^‡^
Inconclusive	0.180	[ 0.116, 0 .258]	0.311	[0.200,0.450]	0.018	[<0.001,0.059]
	1/(LR)^†^					
Identification	0.006	[<0.001, 0.014]	<0.001	[<0.001,0.011]	0.008	[<0.001,0.023]
Elimination	>1000	[121.981, >1000]	>1000	[58.151, >1000]	>1000	[>1000, >1000]^‡^
Inconclusive	5.559	[3.882, 8.657]	3.211	[2.222,5.005]	55.832	[16.863, >1000]

^*^LR values reflect support for the same-source status hypothesis relative to the different-source status hypothesis.

^†^1/(LR) values reflect support for the different-source status hypothesis relative to the same-source status hypothesis.

^‡^0% incorrect decision rate precluded variation in bootstrap estimates.

Note: Median and CI estimates calculated through a multilevel bootstrapping method. Median rather than mean values are reported because even a single bootstrap draw producing an infinite result will cause the mean to be infinite. Bootstrap results that produced the extreme values of 0.000 and infinity are reported as <0.001 and >1000, respectively.

Results showed that rendering a conclusive decision of either identification or elimination provides very strong support for the ground-truth state asserted by that decision, increasing the odds of that ground-truth state more than 100-fold in all cases and for both firearm models. Results also showed that rendering an inconclusive decision provides support for the different-source status hypothesis, increasing the odds of different-source status in all cases and for both firearm models. This tendency is most readily apparent in noting that the lower-limits of the 1/LR CIs for inconclusive decisions always exceeded 1 (or, alternatively, that the upper-limits of the LR CIs for inconclusive decisions never reached 1). An inconclusive decision’s support for the different-source status hypothesis differed by firearm model. For the Beretta comparisons, an inconclusive decision would increase the odds of different-source status by a factor of 3.2, whereas for HiPoint comparisons an inconclusive decision would increase the odds of different-source status by a factor of 55.8. If pretest odds are set at a particular value, these LR results for inconclusive decisions can be converted to posttest probabilities for each source status. For example, if each source status were initially considered to be equally likely for a given comparison, pretest odds would equal one, in which case an inconclusive decision would increase the odds in favor of different-source status such that the posttest probability of the comparison being of different-source status would be 76.3% for Berettas and 98.2% for HiPoints.

## Discussion

This research advances understanding about the validity and probative value of cartridge-case comparison, a commonly used forensic technique. Results based solely on conclusive decisions indicated that cartridge-case comparison is a highly valid forensic technique. Nearly all same-source comparisons received identification decisions, and nearly all different-source comparisons received elimination decisions, causing the accuracy rates pertaining to both the sensitivity and specificity of the technique to be quite high and error rates pertaining to both false-negatives and false-positives to be quite low. These results did not differ across the two firearm models used in the present research, and were similar to those of other rigorous, large-scale validity studies (9, 10). In addition, the current research showed that PPV and NPV were also very high, with identification and elimination decisions strongly predicting same-source and different-source status, respectively, across all but the most extreme prevalence values. The LRs for identification and elimination decisions indicated that conclusive decisions provided very strong support for the ground-truth state asserted by the decision. Specifically, these data suggest that learning that a comparison received either an identification or an elimination decision justifies more than a 100-fold increase in the odds of a comparison’s ground-truth state matching that asserted by the decision.

Consideration of inconclusive decisions, by contrast, necessitates a more nuanced evaluation of the technique’s validity. Inconclusive decisions were not uncommon; in fact, they constituted over one-fifth of all decisions rendered. When using ground truth as the sole criterion for determining correctness, inconclusives are neither correct nor incorrect decisions. Consequently, incorporating inconclusive decisions into calculations caused noticeable reductions in accuracy rates—decreasing specificity (the true-negative rate) more than sensitivity (the true-positive rate). The greater decrement in specificity resulted from inconclusive decisions being rendered more often for different-source than same-source comparisons, a pattern commonly observed in forensic firearm studies (9, 10, 12). Indeed, more than one-third of different-source comparisons received an inconclusive decision, a rate comparable to the 34% observed in one of the prior large-scale validity studies (9), but less than the 51% observed in the other that used comparisons intended to be difficult (10). These findings indicate that either omitting inconclusive decisions or focusing solely on error rates can distort validity assessments by failing to uncover a technique’s reduced ability to yield accurate results. Furthermore, the greater the inconclusive rate, the less the capacity of a technique to achieve what is arguably the foremost purpose of forensic analysis, which is to reveal ground truth.

### Treatment of Inconclusive Decisions.

Current best-practice recommendations for reporting forensic study results, regardless of the particular technique under investigation, include providing detailed data for all conditions and all decisions, conclusive and inconclusive alike (25). Doing so, as we did in Table 1, makes the results maximally useful for the widest range of purposes. However, reporting data for inconclusive decisions then leads to the question of whether they should be incorporated into performance measures for forensic techniques. Considering this question raises several issues. First, the choice to omit inconclusives creates the potential for a self-selection bias, wherein performance measures are based only on the subset of comparisons for which examiners chose to make a conclusive decision, which may not be representative of the full population of comparisons a technique must evaluate (20). Second, the choice to not incorporate inconclusives could obscure effects that influence examiner decisions. For example, the current study showed reduced sensitivity of cartridge-case comparison for evaluations of the more difficult comparisons fired from the Beretta model, but only when incorporating inconclusives in the sensitivity measure calculation. Third, choosing to incorporate inconclusives permits more comprehensive and comparable evaluations of a technique’s validity. For instance, consider two techniques that differ in sensitivity. One technique correctly identifies 100 out of every 100 same-source comparisons examined, whereas the other correctly identifies only 1 out of every 100 same-source comparisons examined, with the remaining 99 receiving inconclusives. Incorporating inconclusives captures this sensitivity difference, whereas omitting inconclusives yields sensitivity estimates of 100% for both techniques, even though one is clearly better at correctly identifying same-source comparisons.

The foregoing points notwithstanding, there may be instances in which it would be apt to omit inconclusives in the calculation of performance measures. For example, one may wish to compare examiner proficiency across studies that have markedly different inconclusive rates as a result of study differences in some other factor, such as comparison difficulty. Omitting inconclusives in this kind of situation may provide a more even-handed comparison of examiner performance by minimizing the effect of factors less related to examiners’ characteristic abilities. Accordingly, the current research reported performance measures from calculations that both omitted and incorporated inclusive decisions. This approach allows one to easily see the differences that stem from various treatments of inconclusive decisions and is consistent with current recommendations (25).

### Determination of Decision Correctness.

We used factual ground truth as the sole criterion for assessing decision correctness because it best matches the key admissibility criterion of relevance as delineated in the Federal Rules of Evidence (26). Specifically, in order to be admitted, evidence (which includes expert testimony) must help “to determine a fact in issue” (27). In a court case, the fact in issue is likewise solely concerned with ground truth, such as whether two pieces of physical evidence are truly from the same source or truly from different sources. For this reason, using ground truth as the sole criterion for determining correctness yields results that are maximally consistent with the purpose the technique will serve in court, and thus most germane to a judge’s evaluation of the technique’s validity and probative value. Although other schemes can be used for designating decisions as correct or incorrect, at best they entail admixtures of ground truth with some subjective determination, such as a decision rule, a consensus opinion, or a similarity measure and its cutoff criterion (18). Evaluations of a technique based on such designations may be useful in some contexts, such as training or evaluating the appropriateness of examiners’ decisions vis-à-vis evidence quality, but they are less useful for assessing how well decisions resulting from application of a forensic technique can determine case facts in court.

### Effect of Firearm Model.

In the current research, restricting analyses to conclusive decisions yielded similar accuracy and error rates across the firearm models. However, differences emerged when incorporating inconclusive decisions. In particular, the sensitivity rate became significantly less for Berettas than HiPoints, presumably because of the models’ dissimilar toolmarks which cause differences in comparison difficulty. HiPoints produce numerous parallel striations that afford many opportunities to confirm agreement. By contrast the toolmarks characteristic of Berettas may less often provide sufficient instances to confirm toolmark agreement, thereby increasing inconclusive decisions and reducing sensitivity. Similarly, LR results showed that inconclusive decisions more strongly supported different-source status for HiPoints than for Berettas. Taken together, these findings highlight the importance of considering whether a technique can be accurately characterized under all conditions present in the field by a single set of performance values, particularly when accounting for the effect of inconclusive decisions.

### Inconclusive Decisions and Ground Truth.

Results of this research showed that inconclusive decisions occurred much more frequently among different-source than same-source comparisons. If decisions had been made solely on the basis of toolmark similarity, classic decision-making models predict that inconclusive decisions would have been more evenly distributed across the ground-truth states (12, 23, 28). One possible cause of the observed asymmetry is that it could be an easier cognitive task to perceive toolmark agreement sufficient to render an identification decision than to perceive toolmark disagreement sufficient to render an elimination decision. However, the greater different-source inconclusive rate could also stem from application of different criteria for making identification versus elimination decisions. Based on the assumption of uniqueness (33), an identification can be justified solely on the basis of toolmark agreement; if toolmarks sufficiently agree, then a single firearm must have fired both cartridge cases. However, there is no corresponding principle that justifies an elimination solely on the basis of toolmark disagreement because a variety of other factors (e.g., intentional alteration, changes with use, presence of debris, variation in ammunition characteristics) could conceivably cause a single firearm to produce dissimilar toolmarks (10).

The above reasoning may cause some examiners to refuse to make elimination decisions as a means to avoid false-negative errors (14). Similar reasoning may underly the policy of some laboratories to prohibit elimination decisions solely on the basis of toolmarks resulting from firearms’ individual characteristics, regardless of how much disagreement is observed (8, 24). These possibilities are consistent with the observation that 18% of examiners in the current study never made an elimination decision. These examiners made only inconclusive decisions for different-source comparisons, but made no inconclusive decisions for same-source comparisons, instead making only identification decisions. This practice seems inconsistent with the decision criteria implied by the Association of Firearm and Tool Mark Examiners (AFTE) range of conclusions scale, which defines the relevant inconclusive category as “Inconclusive with disagreement of individual characteristics but insufficient for elimination” (5). Selecting this decision category implies that the disagreement of individual characteristics could have been sufficient for elimination, when in fact elimination was never possible regardless of the amount of disagreement of individual characteristics.

The practice of eschewing elimination in favor of inconclusive likely contributed to the finding that inconclusive decisions occurred six times more frequently for different-source than same-source comparisons. Consequently, inconclusive decisions predicted different-source status, suggesting that they possess probative value even though they do not assert a ground-truth state. The strength of their probative value as estimated by their LR indicated that an inconclusive decision led to a fivefold increase in the odds of a comparison being different source. The LR result was especially dramatic for HiPoint comparisons wherein the odds increase by a factor of 55.8. These data indicate that inconclusive decisions are useful for determining the fact in issue in criminal proceedings and, therefore, may pass the test for admissibility as delineated in the Federal Rules of Evidence (26, 27).

The frequent rendering of inconclusive decisions for different-source comparisons has serious justice implications. Because an innocent person’s gun is not likely to be the gun used in a crime, actual innocence is associated with different-source status. Therefore, innocent suspects have a substantial likelihood of receiving an inconclusive decision. However, laypeople may interpret inconclusive results to mean that the evidence is thoroughly uninformative, and accordingly fail to realize the exculpatory value of the decision (34). Thus, rendering inconclusive decisions for different-source comparisons could disadvantage the innocent, who may require an unambiguously accurate forensic result to free them from suspicion or to secure an acquittal (35). Accordingly, it is critical for decision makers in the justice system to appreciate the exculpatory value of inconclusive decisions.

## Conclusion

The results of this large sample field study indicated that cartridge-case comparison is a valid forensic technique characterized by conclusive decisions that are rarely in error and, hence, highly accurate in predicting ground truth. However, a substantial proportion of all comparisons received inconclusive decisions, which markedly reduced sensitivity and specificity when incorporated into performance measure calculations. Inconclusive decisions occurred more frequently for different-source status comparisons. Consequently, inconclusive decisions possessed probative value, in that they predicted different-source status, the ground-truth state associated with factual innocence.

## Materials and Methods

*SI Appendix* provides additional methodological detail. The Institutional Review Board at Iowa State University approved the study protocol for this research.

### Participants.

Participants included 228 trained firearm examiners employed in crime labs across the United States. *SI Appendix*, Table S1 provides examiner demographic information.

### Materials.

#### Firearms.

Firearms included 14 Beretta 92FS and 14 HiPoint C9 9-mm pistols. We selected these models because they produce dissimilar toolmarks. Berettas leave pronounced firing pin flowback whereas HiPoints leave linear breech face marks (*SI Appendix*, Figs. S5 and S6). Berettas tend to produce more difficult cartridge-case comparisons than do HiPoints. All firearms had been in use in the general population and were in possession of the Iowa Department of Criminal Investigations.

#### Fired cartridge cases.

We used Winchester 9-mm 115 grain full metal (brass) jacket ammunition, a common ammunition that is readily available to the public for purchase.

### Measures.

#### AFTE Range of Conclusions Scale.

Examiners reported decisions by selecting one of the categories of the AFTE Range of Conclusions Scale (5). We collapsed the inconclusive response categories into a single inconclusive response when analyzing the data.

### Kit Preparation.

Each examiner received four Beretta and four HiPoint comparisons. Each comparison consisted of three known cases that had all been fired from a single firearm, plus either one questioned case that had been fired from the same firearm as the known-cases (a same-source comparison) or one questioned case that had been fired from a different firearm than the known cases (a different-source comparison). Same-source comparisons comprised 50.3% of the total comparisons evaluated, though individual examiners received between three and five same-source comparisons, the exact number being randomly determined. Cartridge cases fired from any particular firearm were used in no more than one of the comparisons evaluated by any given examiner, corresponding to an open-set design.

### Procedures.

Examiners received and returned study materials by mail, including a consent form, examination kit, and response booklet. Examiners returned consent forms in separate mailings for confidentiality reasons.

## Supplementary Material

Appendix 01 (PDF)

## Data Availability

Integer data have been deposited in Harvard Dataverse (https://doi.org/10.7910/DVN/5IYY1Q) (36).
