# Supplementary material for: Validity of forensic cartridge-case comparisons
Source: Proc Natl Acad Sci U S A. 2023 May 8;120(20):e2210428120. doi: 10.1073/pnas.2210428120 (PMC10193974; doi:10.1073/pnas.2210428120)
Supplement: Supplementary file 1 — Appendix 01 (PDF) [file pnas.2210428120.sapp.pdf]

PNAS

Supplementary Information for

Validity of Forensic Cartridge-Case Comparisons

Authors

Max Guyll, Stephanie Madon, Yueran Yang, Kayla A. Burd, Gary Wells

Corresponding Author

Max Guyll

Email: guyll@asu.edu

This PDF file includes:

Supplementary text

Figures S1 to S6

Table S1

SI References

Other supplementary materials for this manuscript include the following:

None

Materials and Methods

The Institutional Review Board at Iowa State University approved the study protocol for this research.

Participants

Participants included 228 trained firearm examiners employed in private, municipal, county, state and federal crime labs in the United States. Full examiner characteristic information appears in SI Appendix Table S1.

Materials

Firearms. The Iowa Department of Criminal Investigation (Iowa DCI) provided 14 Beretta model 92FS and 14 HiPoint model C9 9mm pistols for the purpose of producing the cartridge-case specimens. We selected these two firearm models because they differ with respect to the toolmarks typically transferred to the cartridge-case. For example, Beretta 92FS pistols leave pronounced firing pin flowback on the cartridge-case primer, whereas HiPoint C9 pistols leave a large number of linear breech face marks on the cartridge-case head (see SI Appendix Figures S5 and S6). Beretta's produce cartridge-case comparisons that are more difficult than those produced by HiPoint. The 28 firearms had been in circulation in the general population.

Fired cartridge-cases. We used Winchester 9mm 115 grain full metal (brass) jacket ammunition to produce the fired cartridge-cases for examination, a common ammunition that is readily available for purchase by the general population. Firing of ammunition took place at an indoor range during reserved times. Only Iowa DCI firearm examiners handled the firearms. Mesh cuboid-shaped cartridge-case catchers caught the fired cartridge-cases as they were ejected from the firearm. All cartridges cases that came to rest anywhere other than the inside of the catcher were discarded. Research team members provided support by loading magazines and affixing labels to identify the firearm used to fire the collected cartridge-cases.

Measures

AFTE Range of Conclusions Scale. For each cartridge-case comparison performed, examiners reported a forensic decision by selecting one of the categories corresponding to the

AFTE Range of Conclusions Scale: Identification, Elimination, Inconclusive without further characterization, Inconclusive with some agreement of individual characteristics but insufficient for identification, Inconclusive without agreement or disagreement of individual characteristics, Inconclusive with disagreement of individual characteristics but insufficient for elimination, and Unsuitable for analysis (1). We collapsed responses across the four inconclusive categories to create a single inconclusive response.

Kit Preparation

Each examiner received a unique examination kit. Each kit included eight comparisons of fired cartridge-cases, including four produced by Beretta pistols and four produced by HiPoint pistols. Within a given kit, no single firearm produced cartridge-cases for use in more than a single comparison. This feature of the research corresponds to an open set design that required examiners to make independent forensic decisions on each comparison evaluated. Each comparison consisted of three known cases that had all been fired from a single firearm, plus either one questioned case that had been fired from the same firearm as the known-cases (a same-source comparison), or one questioned case that had been fired from a different firearm than the known cases (a different-source comparison). Each kit contained between three and five same-source comparisons. Each firearm model produced between one and three same-source comparisons. The particular number of same-source and different-source comparisons varied randomly across examiners. Across all examiners who participated in the study, the rate of same-source comparisons equaled 50.3%. Examiners were unaware of these parameters.

Procedures

The research team mailed each examiner an examination kit, response booklet, and consent form. The response booklet assessed demographic and professional information (SI Appendix Table S1), provided detailed instructions, and included eight reporting forms, one for each of the eight comparisons, on which examiners recorded their forensic decisions. Examiners returned all study materials to the research team using prepaid mailing envelopes. Consent forms were returned in separate mailings for confidentiality reasons.

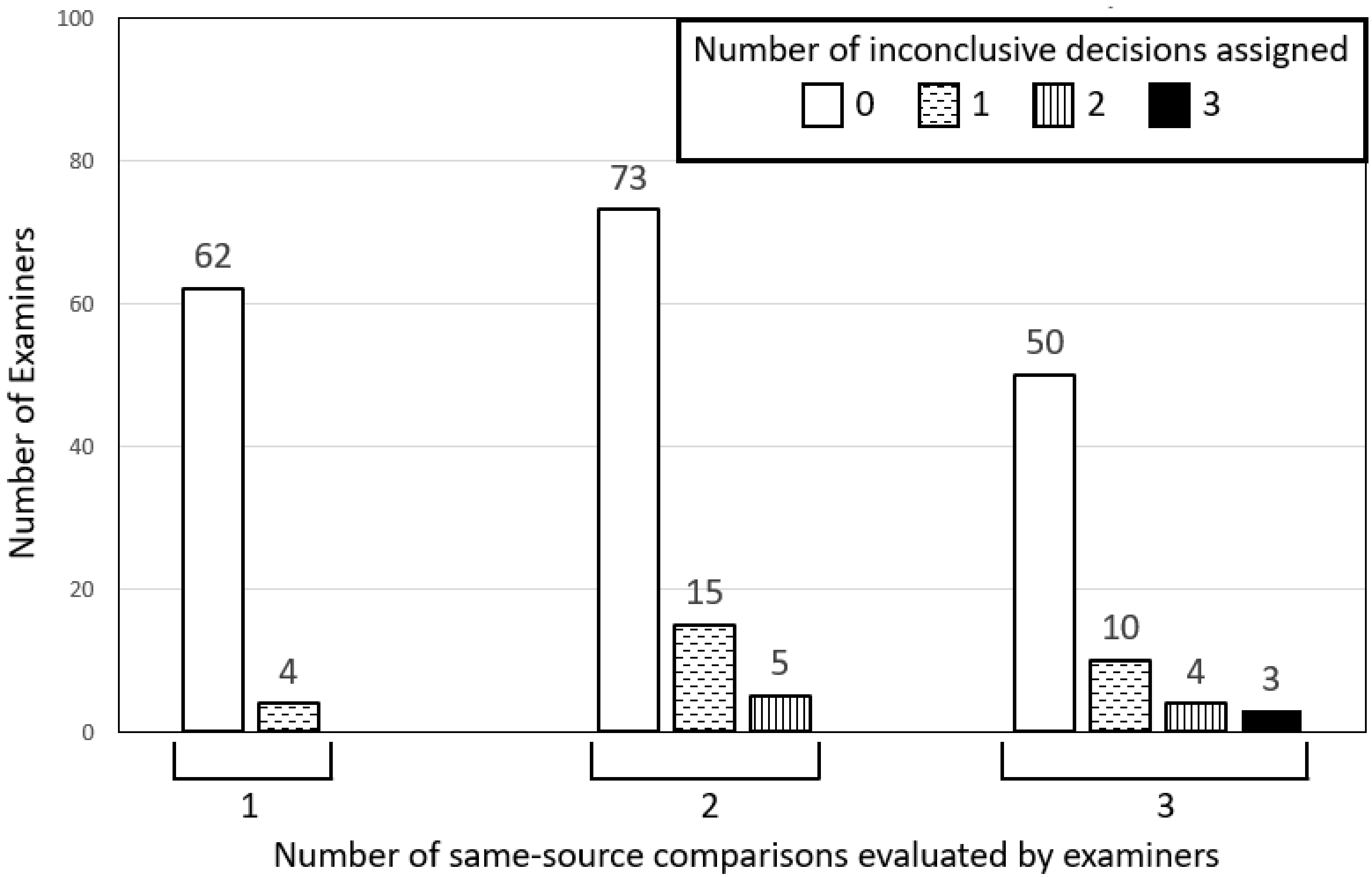

Fig. S1. Number of examiners assigning various numbers of inconclusive decisions for Beretta 92FS same-source comparisons.

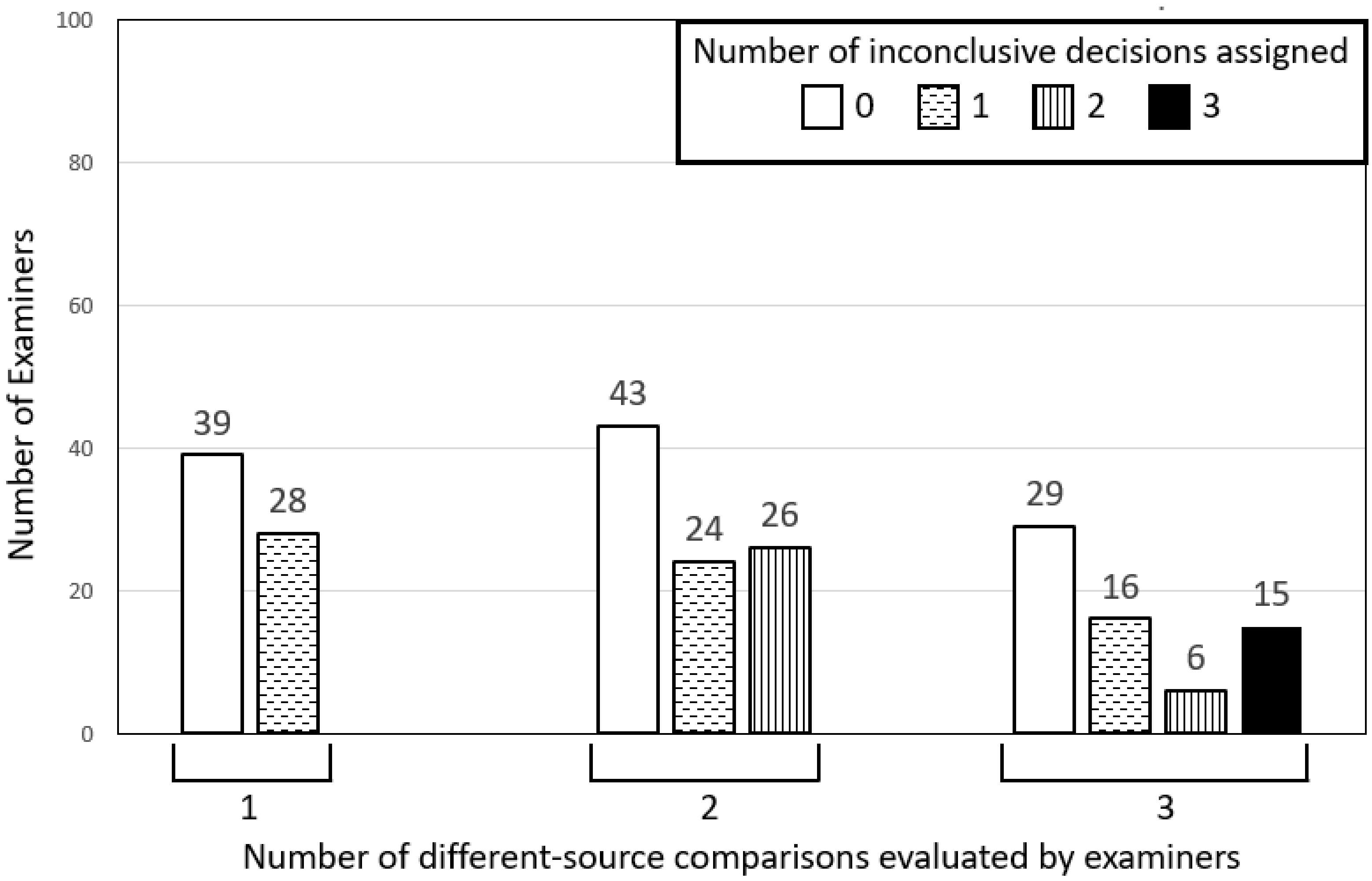

Fig. S2. Number of examiners assigning various numbers of inconclusive decisions for Beretta 92FS different-source comparisons.

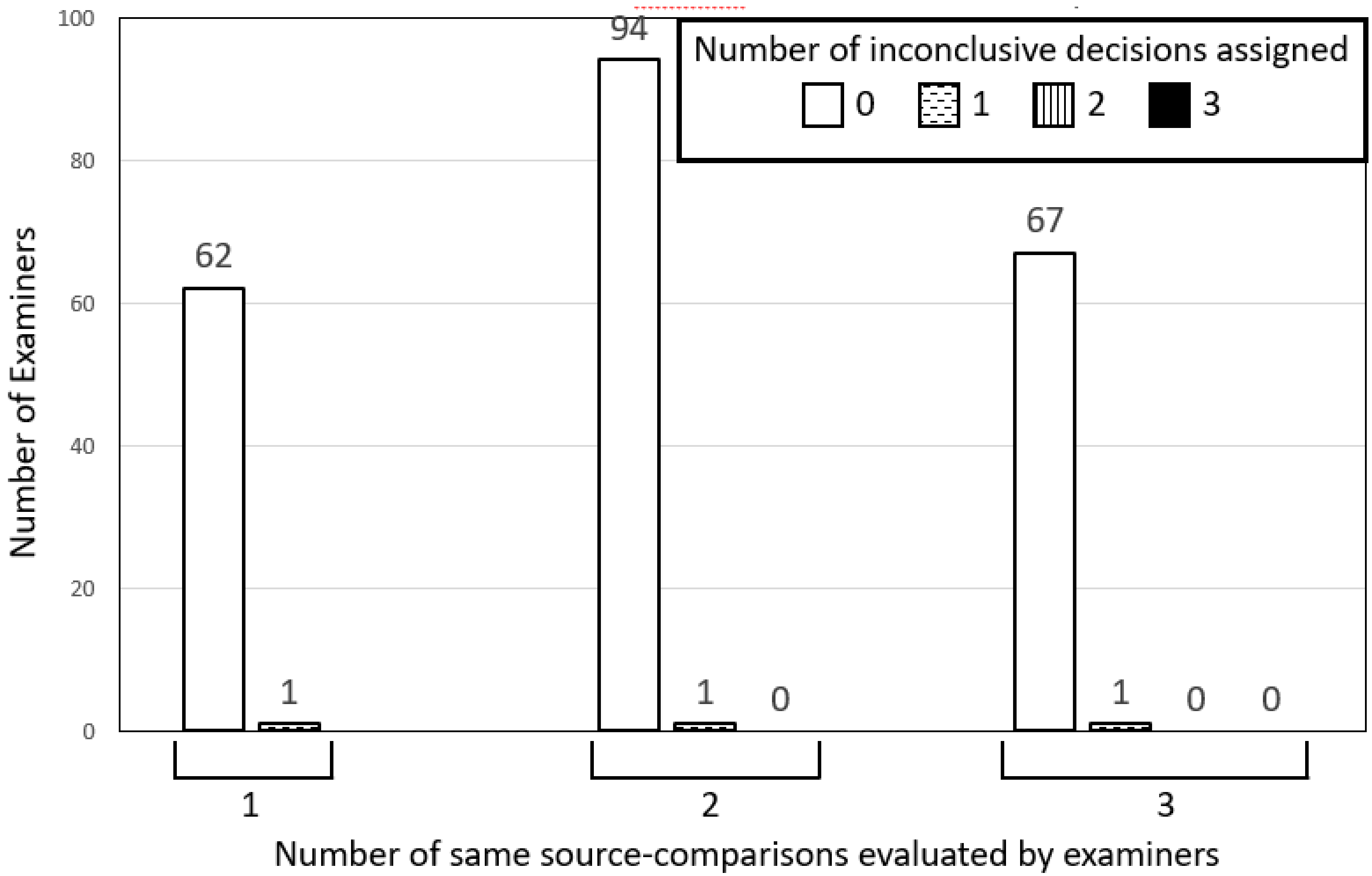

Fig. S3. Number of examiners assigning various numbers of inconclusive decisions for HiPoint C9 same-source comparisons.

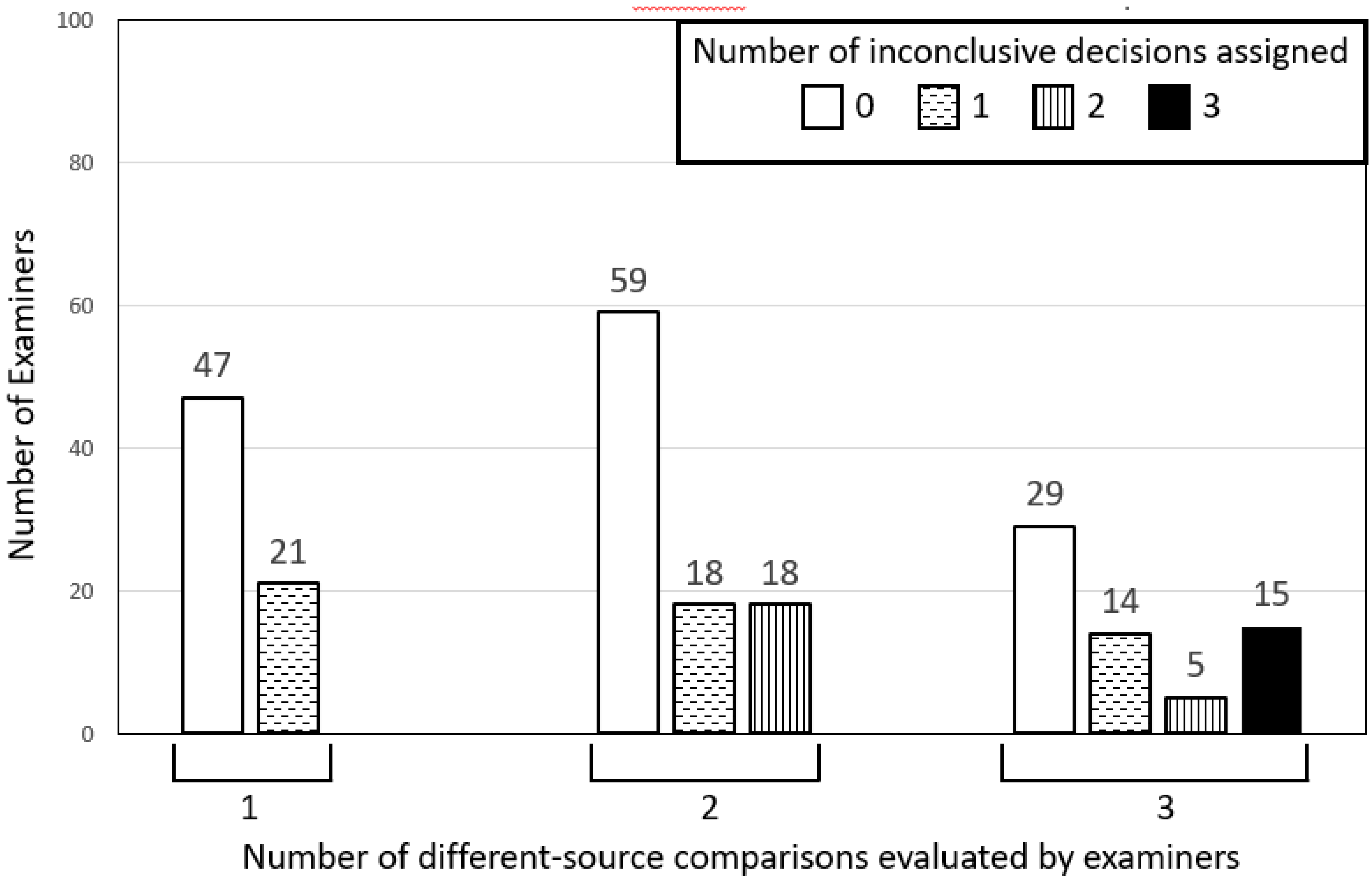

Fig. S4. Number of examiners assigning various numbers of inconclusive decisions for HiPoint C9 different-source comparisons.

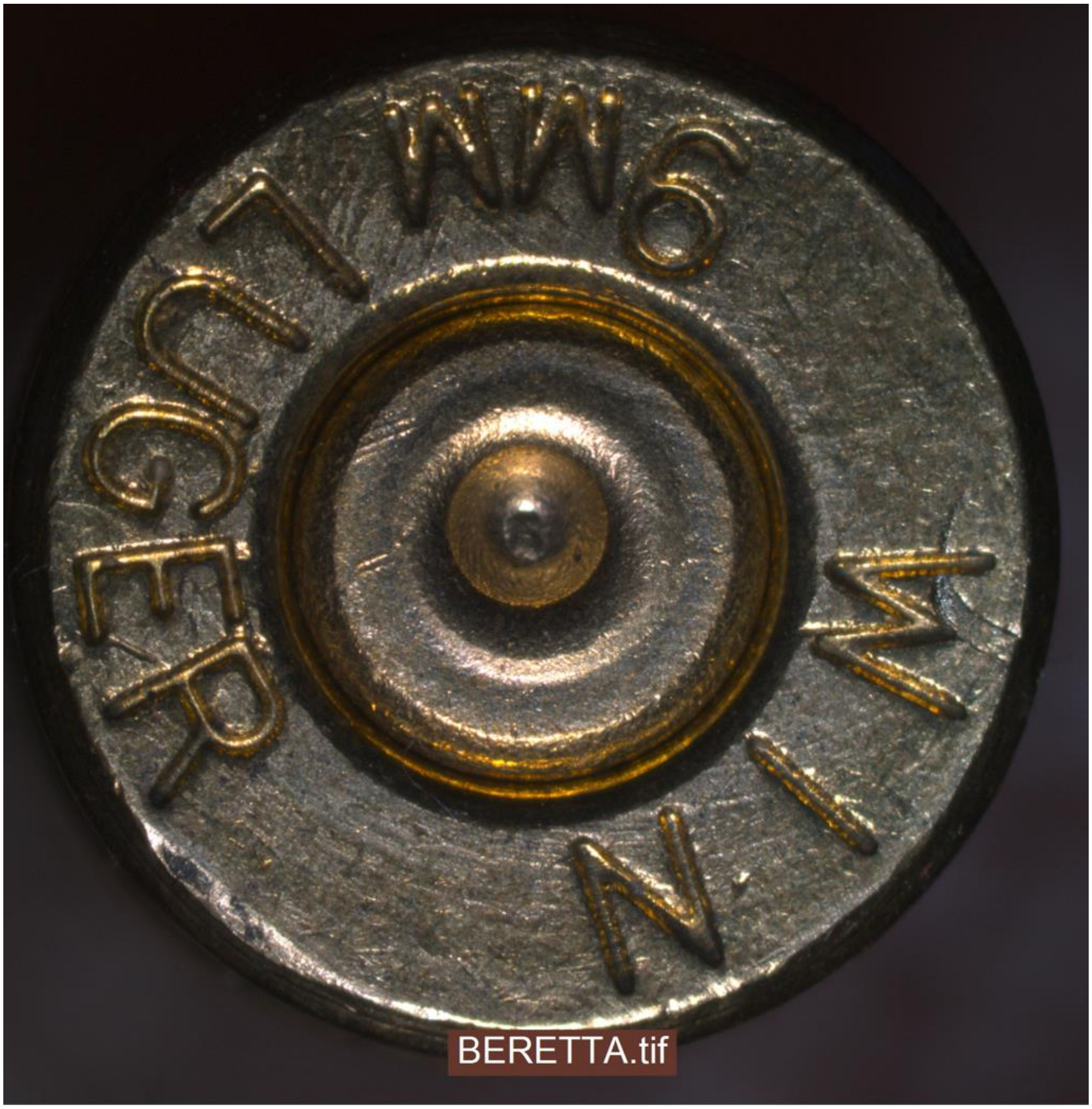

Fig. S5. Typical toolmarks on cartridge-case fired by a Beretta 92FS.

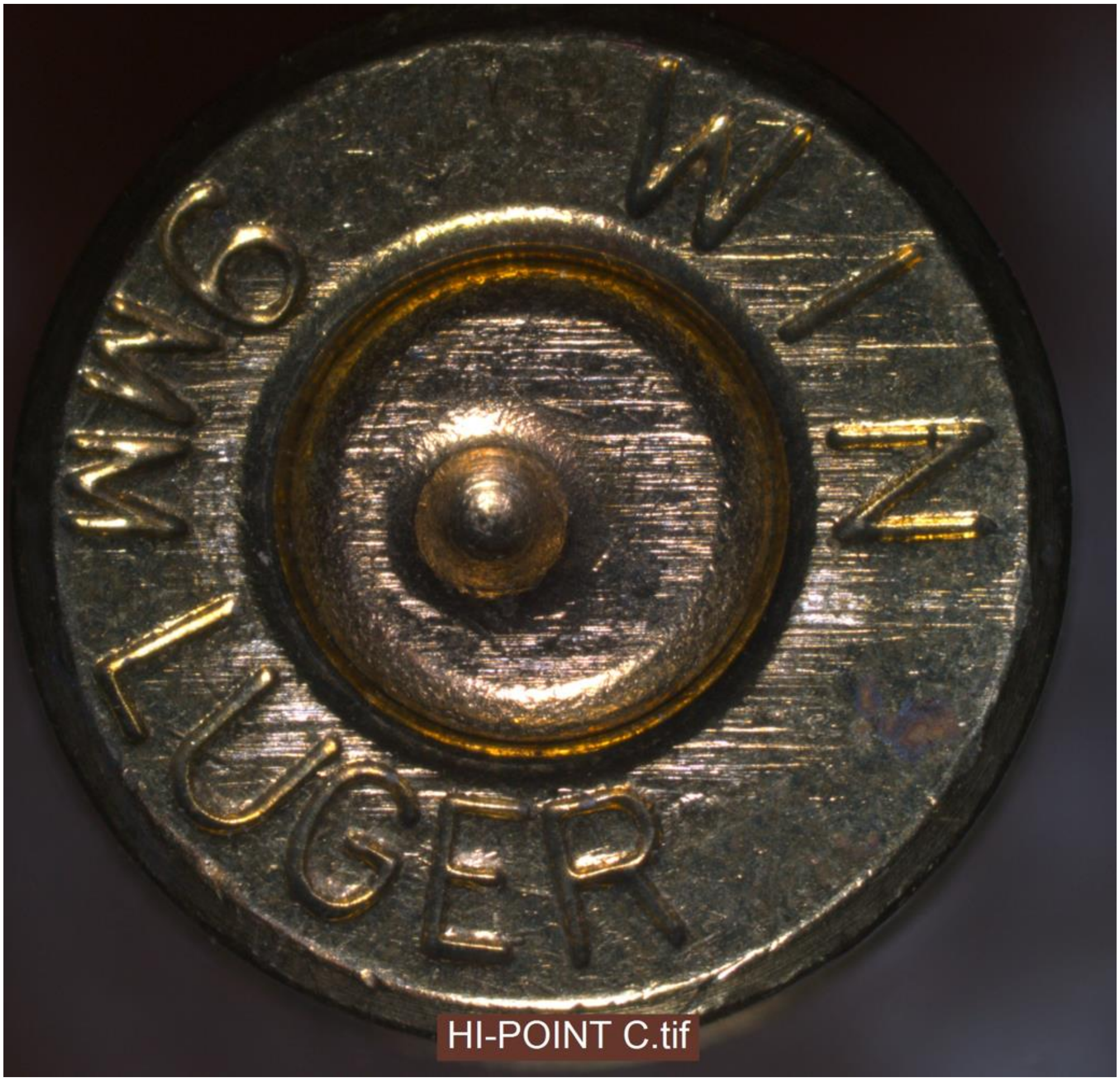

Fig. S6. Typical toolmarks on cartridge-case fired by a HiPoint C9.

Table S1. Examiner descriptive information (*N* = 228).

Training (years)[a]	*mdn* =	2.0 (1.0; 2.0)
Experience (years)[b]	*mdn* =	9.4 (4.4; 16.0)
Continuing education (hours, past year)[c]	*mdn* =	0.0 (0.0; 10.0)
Age (years)[d]	*mdn* =	40 (33; 48)
AFTE member[e]		87.2%
AFTE certified[e]		26.0%
ABC certified[e]		4.0%
CMS trained[e]		34.4%
FBI Specialized Techniques School[e]		7.9%
ANAB member laboratory[f]		88.4%
Comparison method used[b]		
Pattern matching only		84.1%
Continuous matching striae only		0.0%
Both		15.9%
Laboratory elimination decision policy[b]		
Permitted on basis of class characteristics		96.5%
Permitted on basis of individual characteristics		91.2%
Elimination decisions not permitted		0.0%
Education[d]		
High school diploma or GED		1.8%
Two-year college degree		1.8%
Four-year college degree		62.1%
Master's degree		33.9%
Doctoral degree		0.4%
Sex/Gender[g]		
Female		40.1%

Male	59.9%

Note. *mdn* = median. Parenthetical values are 1st and 3rd quartiles. AFTE = Association of Firearm and Tool Mark Examiners. ABC = American Board of Criminalists. ANAB = American National Standards Institute National Accreditation Board. CMS = Consecutive Matching Striae. [a]*n* = 223 reporting. [b]*n* = 226 reporting. [c]*n* = 205 reporting. [d]*n* = 224 reporting. [e]*n* = 227 reporting. [f]*n* = 225 reporting. [g]*n* = 217 reporting.

SI References

1. AFTE Criteria for Identification Committee, Theory of Identification, range striae comparison reports and modified glossary definitions. *AFTE J.* **24,** 336-340 (1992).
